# Volumetric reconstruction and determination of minimum crosssectional area of the pharynx in patients with cleft lip and palate: comparison between two different softwares

**DOI:** 10.1590/1678-7757-2017-0282

**Published:** 2018-10-02

**Authors:** Maycon Lázaro Pinheiro, Marília Yatabe, Marcos Ioshida, Luan Orlandi, Priscille de Dumast, Ivy Kiemle Trindade-Suedam

**Affiliations:** 1 Universidade de São Paulo, Faculdade de Odontologia de Bauru, Bauru, São Paulo, Brasil; 2 Universidade de São Paulo, Hospital de Reabilitação de Anomalias Craniofaciais, Bauru, São Paulo, Brasil; University of Michigan, School of Dentistry, Ann Arbor, United States of America; Universidade de São Paulo, Hospital de Reabilitação de Anomalias Craniofaciais, Bauru, São Paulo, Brasil; University of Michigan, School of Dentistry, Ann Arbor, United States of America; 3 Universidade de São Paulo, Instituto de Ciências Matemáticas e de Computação, São Carlos, São Paulo, Brasil; 4 University of Michigan, School of Dentistry, Ann Arbor, United States of America; 5 Universidade de São Paulo, Faculdade de Odontologia de Bauru, Departamento de Ciências Biológicas; Hospital de Reabilitação de Anomalias Craniofaciais, Laboratório de Fisiologia, Bauru, São Paulo, Brasil

**Keywords:** Cone-beam computed tomography, Three-dimensional imaging, Pharynx, Oropharynx

## Abstract

**Objective:**

The aim of this study was to assess the accuracy of volumetric reconstruction of the pharynx by comparing the volume and minimum crosssectional area (mCSA) determined with open-source applications (ITK-Snap, www.itksnap.org ; SlicerCMF) and commercial software (Dolphin3D, 11.8, Dolphin Imaging & Management Solutions, Chatsworth, CA, USA) previously validated in the literature.

**Material and Methods:**

The sample comprised of 35 cone-beam computed tomography (CBCT) scans of patients with unilateral cleft lip and palate, with mean age of 29±15. Three-dimensional volumetric models of the pharynx were reconstructed using semi-automatic segmentation using the applications ITK-Snap (G1) and Dolphin3D (G2). Volumes and minimum cross-sectional areas were determined. Inter- and intra-observer error were calculated using ICC test. Comparison between applications was calculated using the Wilcoxon test.

**Results:**

Volumes and minimum crosssectional area were statistically similar between applications. ITK-Snap showed higher pharynx volumes, but lower mCSA. Visual assessment showed that 62.86% matched the region of mCSA in Dolphin3D and SPHARM-PDM.

**Conclusion:**

Measurements of volume and mCSA are statistically similar between applications. Therefore, open-source applications may be a viable option to assess upper airway dimensions using CBCT exams.

## Introduction

From a physiological point of view, 60% of the individuals with cleft lip and palate present compromised upper airway due to nasal septum deviation, nasal turbinate hypertrophy, and nasal floor alterations. ^1^ These changes reduce the internal dimensions of the nasal cavity, increase resistance to respiratory airflow, and, for a considerable amount of individuals, it produces oral breathing, which can impair craniofacial development, and compromise the function of airways and speech. ^2^ Studies comparing lateral cephalometric radiographs of children with and without clefts found an association between the significant reduction of pharyngeal dimensions and the retro-positioned maxilla in children with cleft, leading to a reduction of the skeletal nasopharynx and consequently of the pharyngeal air space. ^3^ ^,^ ^4^ However, lateral radiographs represent only two dimensions of a three-dimensional structure, and therefore offers limited information regarding the airways. ^5^

Cone-beam computed tomography (CBCT) has become a popular method to diagnose and visualize upper airways due to its relatively low cost, less radiation dose (compared to traditional computed tomography), and better accuracy in identifying the limits between soft and hard tissues. ^6^ In addition, information on crosssectional areas and volumes can only be determined by three-dimensional images. ^6^ ^,^ ^7^ Up to date, no consensus regarding pharyngeal dimensions in the population of individuals with cleft lip and palate has been achieved. Some authors suggest that the pharynx of non-operated individuals with cleft palate is volumetrically larger than individuals without clefts, ^8^ while others suggest that total airway volumes of operated patients with unilateral cleft are smaller. ^3^ ^,^ ^4^ ^,^ ^9^ There is also a third group of authors who suggest there are no dimensional differences between the posterior air space of individuals with and without cleft lip and palate. ^10^ ^,^ ^11^ The different results obtained might be related to the divergence in selection criteria of their corresponding samples, as well as the methodologies used.

Recently, segmentation techniques have been available to the clinical researcher. Among different applications, it may variate from manual to automatic segmentation, and requires prior knowledge about the form and intensity of the structures of interest. The semi-automatic technique combines the accuracy, high efficiency and repeatability of the automatic methods with the experience and quality control of an operator's supervision. ^12^ ^,^ ^13^ The commercial application software Dolphin3D (11.8, Dolphin Imaging & Management Solutions, Chatsworth, CA, USA) is widely used and validated in the literature for assessing upper airway images obtained from cone-beam computed tomography. ^3^ ^,^ ^4^ ^,^ ^11^ ^,^ ^14^ ^-^ ^19^ Among free software for threedimensional assessments, ITK-SNAP ( www.itksnap.org ) was developed to segment volumetric models in a simplified way for users without mathematical knowledge, ^12^ ^,^ ^13^ and SPHARM-PDM module was developed for the free application software SlicerCMF ( www.slicer.org ) to create parametric models based on harmonic spheres. Given this context, the aim of this study was to verify the accuracy of a free, open-source application in three-dimensional upper airway assessment, determining volume and minimum cross-sectional area (mCSA), compared to a commercial application software previously validated in the literature. It is our hypothesis that pharyngeal airway measurements are similar in both applications.

## Material and methods

The Research Ethics Committee of the Hospital for Rehabilitation of Craniofacial Anomalies, University of São Paulo approved this retrospective study (CEP: 15205413.7.0000.5441).

The initial sample comprised 69 concomitants CBCT exams from the Laboratory of Physiology - Hospital for Rehabilitation of Craniofacial Anomalies, University of São Paulo. Inclusion criteria were: patients with unilateral complete cleft lip and palate with a malocclusion of Angle Class III, and CBCT scans with a field of view of at least 13 cm. Exclusion criteria were: scans with incompatible format with the software, and poor image quality. The final sample consisted of 35 exams of patients with unilateral cleft lip and palate, with a mean age of 29±15.

**Figure 1:**
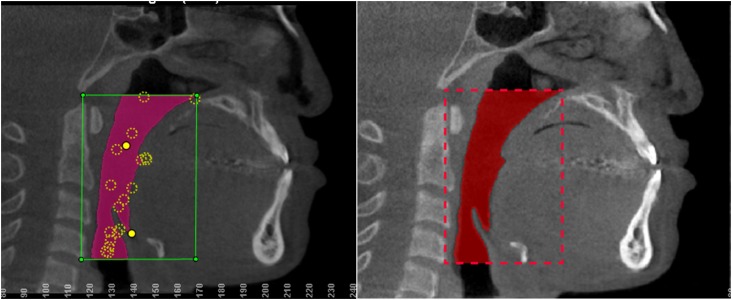
Rectangle formed by the selected anatomic points. On the left, image from Dolphin3D software and, on the right, image from ITK-Snap software

**Figure 2:**
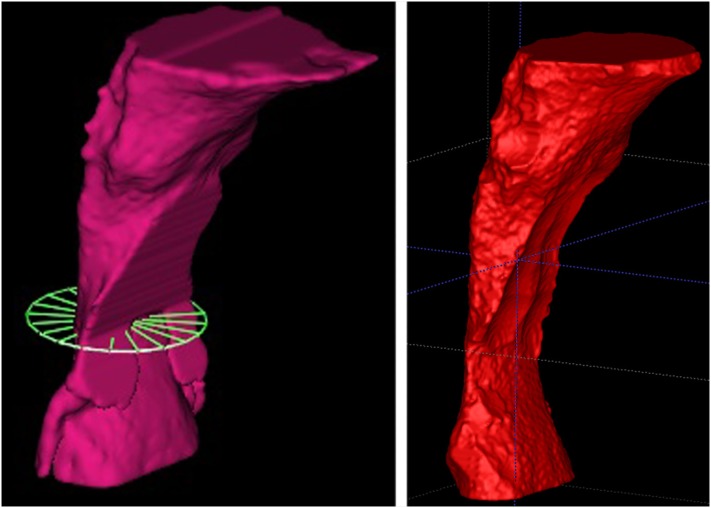
Segmentation of a single patient obtained with Dolphin3D (left) and with ITK-Snap (right)

For determination of the pharyngeal volume, threedimensional volumetric models were constructed and measured using two applications: Dolphin3D (11.8, Dolphin Imaging & Management Solutions, Chatsworth, CA, USA) and ITK-Snap ( www.itksnap.org ). ^12^ ^,^ ^13^ Anatomical points defined the boundaries of the region of interest: antero-inferior border of the fourth cervical vertebra (C4), lower-posterior border of the hyoid bone, anterior pharyngeal wall and basal, forming a rectangle ( Figure 1 ). The grayscale threshold determined for the airway segmentation was individualized for each scan, and similar for both applications. Figure 2 shows the pharyngeal segment from a single patient reconstructed using the Dolphin3D and ITK-Snap applications.

**Figure 3:**
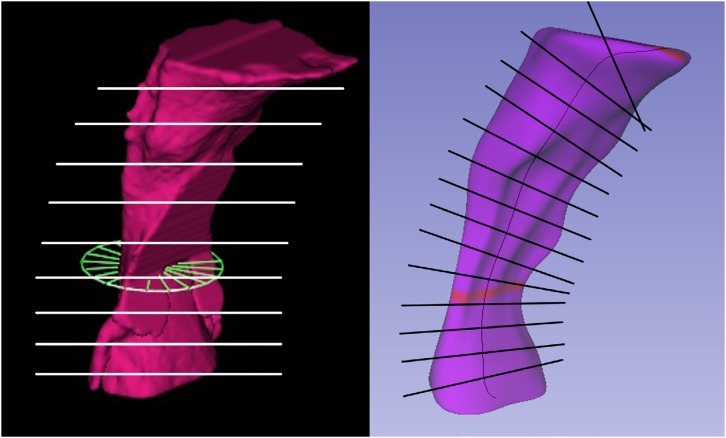
Mean axis and highlighted mCSA obtained with Dolphin3D (left) SPHARM-PDM module (right)

To determine mCSA, “bubbles” and spicules were removed from the original segmentation, leaving the final three-dimensional model with a cylindrical and smoother surface. With these changes, the epiglotic vallecula, which participates in the original segmentation, had to be excluded. The mCSA was automatically determined by Dolphin3D. As for the free software, the volumetric models segmented on ITK-SNAP ^12^ ^,^ ^13^ were exported to another open-source software: SlicerCMF with SPHARM-PDM module. In this module, parametric surface models were created, as well as a mean axis of the volume, in order to determine mCSA ( Figure 3 ).

**Figure 4:**
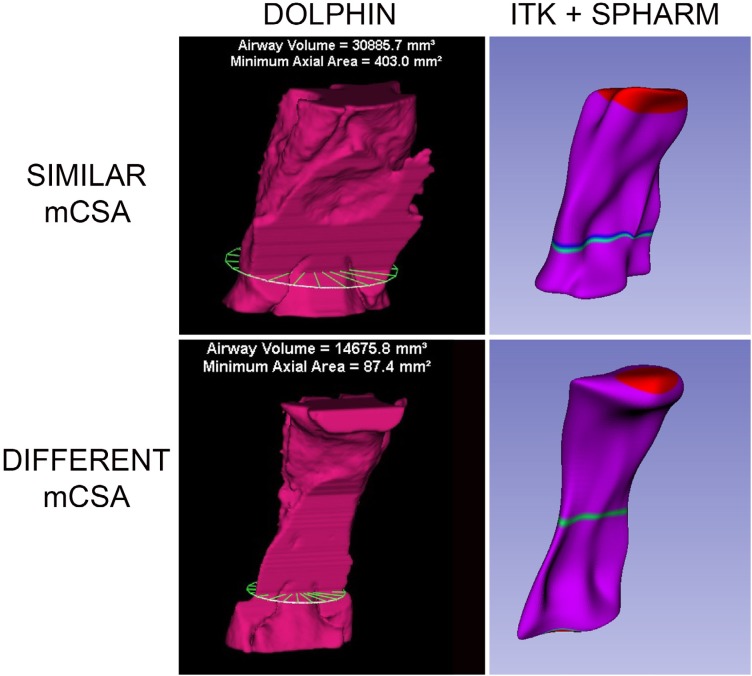
Models with similar and different position of the mCSA between segmentations performed in Dolphin3D (left) and ITK-Snap (right)

Visual analysis of the position of the minimum crosssectional areas between the volumetric models obtained by the two applications was performed. The models were compared and organized into two groups: position of a similar cross-sectional area, and position of different cross-sectional area ( Figure 4 ).

To determine intra- and inter-observer error, 30% of the sample was re-evaluated by two examiners previously calibrated with a 30-day interval, as well as visual analysis.

### Statistical analysis

ICC test was used to calculate inter- and intraobserver agreement. Normality distribution was calculated using the Shapiro-Wilk test, and to compare the volumes and mCSA, paired *t-test* and Wilcoxon test were applied for normal and non-normal distribution, respectively.

## Results

Inter- and intra-observer correlation tests presented high agreement, varying from 0.91 to 1.00. Only the mCSA from the Dolphin Software showed a normal distribution, therefore only the Wilcoxon test was conducted for all statistical analysis.

The mean volume of the three-dimensional volumetric models was higher in the ITK-Snap software, however no significant difference (p>0.05) was observed. On the contrary, the mean value of the minimum cross-sectional area was lower in the ITK-Snap software, but the difference was also not statistically significant (p>0.05) ( Table 1 ).

**Table 1:** Volume and minimum cross-sectional area values obtained by the applications Dolphin3D, ITK-SNAP, and SlicerCMF with SPHARM-PDM module

		Volume (cm ^3^ )		Minimum cross-sectional area (mm ^2^ )	
EXAM	AGE	ITK-SNAP	Dolphin3D	ITK-SNAP, SlicerCMF (SPHARM-PDM module)	Dolphin3D
1	25	27.63	28.60	1.47	1.26
2	27	28.56	32.50	1.49	1.90
3	25	20.26	19.38	1.11	0.96
4	21	14.15	16.60	0.80	1.04
5	23	20.57	22.67	2.24	1.39
6	23	40.52	35.54	4.41	4.03
7	30	9.93	8.85	0.49	0.38
8	19	23.58	22.88	2.34	1.43
9	22	18.16	17.09	1.51	0.87
10	25	25.86	26.88	1.45	1.45
11	28	16.64	16.70	1.17	1.29
12	40	14.65	16.40	1.13	0.46
13	18	19.50	17.58	1.44	1.39
14	22	20.43	17.32	1.28	1.17
15	24	22.17	22.19	1.32	1.40
16	37	21.64	22.80	0.65	0.80
17	25	9.76	14.31	0.64	0.85
18	33	19.06	21.29	1.56	1.06
19	26	15.86	15.67	1.63	1.14
20	19	24.74	28.51	1.81	1.55
21	26	17.53	19.33	1.53	1.34
22	34	15.10	15.88	1.10	1.13
23	25	11.03	12.01	0.96	0.99
24	24	14.01	16.55	1.10	1.09
25	23	16.19	17.27	1.13	1.12
26	21	15.76	20.71	0.95	0.85
27	23	32.90	31.65	1.52	1.35
28	27	13.60	14.28	0.93	1.09
29	29	22.62	22.85	1.12	1.18
30	50	8.89	11.65	0.42	0.40
31	23	15.02	15.66	1.03	0.99
32	31	17.65	18.24	0.73	0.76
33	54	12.84	13.98	0.80	0.46
34	55	14.85	17.92	0.85	0.44
35	59	16.31	19.63	1.27	0.87
Mean	29	23.57	22.86	1.21	1.86
SD	15	8.52	8.3	0.43	1.22
*p*		*0.66*		*0.14*	

Figure 4 shows two surface models created from Dolphin3D and SPHARM-PDM (SlicerCMF) used for visual analysis of the position of the minimum crosssectional area, divided into similar and different position of the mCSA, respectively.

## Discussion

Cone-beam computed tomography presents several advantages for modern dentistry, such as accurate diagnoses, reliable surgical treatments with more predictable prognoses, lower costs compared to medical tomography. ^20^ The reconstruction of volumetric models is closely related to the functionalities of the CBCT, being fundamental for the success of the tomography insertion in research and dental practice. Dolphin3D (11.8, Dolphin Imaging & Management Solutions, Chatsworth, CA, USA) is a validated software in the literature and widely used for the analysis of CBCT images. ^3^ ^,^ ^4^ ^,^ ^11^ ^,^ ^14^ ^-^ ^19^ However, since it is a high-cost commercial product, it might be inaccessible to independent and clinical researchers. The ITK-Snap ( www.itksnap.org ) is an open source software, and among the advantages of open source software are: the possibility of new updates and/or modifications, good interaction with other applications, and tool customizability. ^21^

In software development, two concepts, “user interface” and “user experience”, are important in determining the ease and experience of the user in using them. The user interface determines the human-machine interface, that is, the interface used to control the software. It includes GUI (Graphical User Interface), the presentation of the interface in graphical elements, buttons, menus and organization of the elements. The more organized and intuitive, the better. These applications work with very specific actions, making it hard to provide a good enough user interface for clinicians, for example. Intimately linked to the intuitiveness and organization of the user interface, it includes the aesthetic appearance of the software, the content presented to the user and the response time of the actions. It has great influence on the choice of software when it needs to be used frequently.

**Figure 5:**
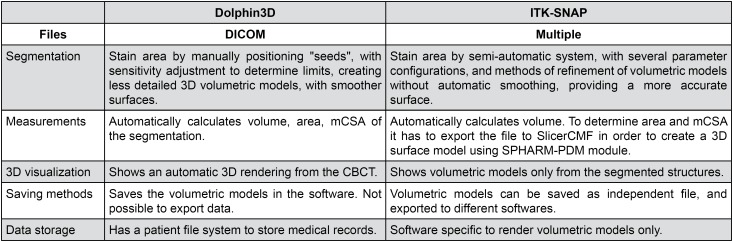
Technical differences found by experiencing both applications

According to El and Palomo ^22^ (2010), the automatic segmentation method, commonly used for the calculation of airway space, is simple, but it fails because of lack of accuracy. They suggest that segmentation should be performed semi-automatically, which means combining automatic and manual segmentation, resulting in better measurement accuracy. Amongst the two applications compared in this study, some technical differences were found, as displayed in Figure 5 . Even though ITK-Snap software is apparently more reliable to the grayscale levels of each voxel of the CBCT exam, resulting in slightly higher volumes of the pharynx, those differences had a minor impact on volume determination after segmentation ( Table 1 ). Thus, it is important to emphasize that pharyngeal volumes obtained with both applications were statistically similar.

Commercial and open-source software showed different methodologies for determining mCSA, as described in Figure 5 . The commercial software Dolphin3D has an automatic function. It determines which axial slice (parallel to the horizontal plane) shows the smallest segmented area. The determination of this mCSA is dependable on head orientation at the moment of segmentation. On the other hand, to determine mCSA using open source software, additional steps are necessary: (1) removal of “bubbles” and spicules from the original segmentation, leaving the final three-dimensional model with a cylindrical and smoother surface. With these changes, the epiglotic vallecula, which participates in the original segmentation, had to be excluded; (2) export the segmentation file to SPHARM-PDM module, in SlicerCMF, another open source software. After running the SPHARM-PDM module, several files are created, including the surface model with a smoother surface, its mean axis and a table with all cross-sectional areas perpendicular to the mean axis ( Figure 2 ).

For comparison purposes of the mCSA, the same changes were applied to the segmentations made in the Dolphin3D software. As observed in Table 1 , the mCSAs found in SPHARM-PDM were smaller when compared to those found with Dolphin3D, but with no statistical difference. Additionally, it was observed that 62% of the models showed similar regions of the mCSA between applications. The difference of mCSA could be related to the fact that Dolphin3D assesses the axial slice parallel to the horizontal plane with the mCSA segmented, independently of the anatomy and position of the skull, whereas SPHARM-PDM assesses the minimal cross-sectional slice perpendicular to the mean axis of the pharynx model ( Figure 4 ), which could make more sense from an anatomical perspective.

## Conclusion

Volume and minimum cross-sectional areas of the pharynx obtained by open source software can be statistically similar to the findings of commercial software. Therefore, open source software may be a viable, free option to assess upper airway dimensions using cone-beam computed tomography exams.
